# Knowledge structure and emerging trends in the application of deep learning in genetics research: A bibliometric analysis [2000–2021]

**DOI:** 10.3389/fgene.2022.951939

**Published:** 2022-08-23

**Authors:** Bijun Zhang, Ting Fan

**Affiliations:** ^1^ Department of Clinical Genetics, Shengjing Hospital of China Medical University, Shenyang, China; ^2^ Department of Computer, School of Intelligent Medicine, China Medical University, Shenyang, China

**Keywords:** deep learning, machine learning, genetics, bibliometric, knowledge graph

## Abstract

**Introduction:** Deep learning technology has been widely used in genetic research because of its characteristics of computability, statistical analysis, and predictability. Herein, we aimed to summarize standardized knowledge and potentially innovative approaches for deep learning applications of genetics by evaluating publications to encourage more research.

**Methods:** The Science Citation Index Expanded ^TM^ (SCIE) database was searched for deep learning applications for genomics-related publications. Original articles and reviews were considered. In this study, we derived a clustered network from 69,806 references that were cited by the 1,754 related manuscripts identified. We used CiteSpace and VOSviewer to identify countries, institutions, journals, co-cited references, keywords, subject evolution, path, current characteristics, and emerging topics.

**Results:** We assessed the rapidly increasing publications concerned about deep learning applications of genomics approaches and identified 1,754 articles that published reports focusing on this subject. Among these, a total of 101 countries and 2,487 institutes contributed publications, The United States of America had the most publications (728/1754) and the highest h-index, and the US has been in close collaborations with China and Germany. The reference clusters of SCI articles were clustered into seven categories: deep learning, logic regression, variant prioritization, random forests, scRNA-seq (single-cell RNA-seq), genomic regulation, and recombination. The keywords representing the research frontiers by year were prediction (2016–2021), sequence (2017–2021), mutation (2017–2021), and cancer (2019–2021).

**Conclusion:** Here, we summarized the current literature related to the status of deep learning for genetics applications and analyzed the current research characteristics and future trajectories in this field. This work aims to provide resources for possible further intensive exploration and encourages more researchers to overcome the research of deep learning applications in genetics.

## 1 Introduction

Deep learning (DL) is a subfield of machine learning (ML) that aims to avoid extensive manual processing in traditional methods Wu et al., (2020). Different from machine learning, deep learning is a form of representation learning in which a machine is fed with raw data and develops its own representations needed for pattern recognition—which is composed of multiple layers of representations Esteva et al., (2019). The application of DL in medical healthcare has been widely reported. For example, DL has been reported to be successful in identifying a variety of histopathological features and detecting the biomarkers (Berrar and Dubitzky, 2021). DL has also been applied to predict diagnosis, prognosis, and treatment response in certain cancers Tran et al., (2021). This information could prove valuable in clinical decision-making for cancer treatment and triage for in-depth sequencing.

Genomic data had served as a biomarker for the onset and progression of the disease. Various deep learning applications in genomics had been reported, such as predicting gene expression from genotype data and studying the splicing-code model and the identification of long noncoding RNAs (Tripathi et al., 2016; Xie et al., 2017; Vellido, 2020; Tang et al., 2021). Recent advances in deep learning have emerged in several applications, ranging from natural language to vision processing Zou et al., (2019). Bibliometric approaches have generated a considerable impact on the deep learning research field, such as deep learning networks in identifying medical images and histopathology images for breast cancer classification (Khairi et al., 2021; Wang et al., 2022). However, gaps exist for deep learning in genetics research, and there is a dearth of information on associated bibliometric development trends. Therefore, based on deep learning technology advancements in genetics, a comprehensive bibliometric overview is required to provide researchers with new future research directions.

In the present study, we accessed the Web of Science Core Collection (WoSCC) using bibliometric methods to review and select deep learning studies in genetics research from 2000 to 2021. Specifically, co-word biclustering analysis was utilized to identify the research hot spots of the application of DL in genomics research. We hope this article can provide some reference for future research on deep learning and genomics research.

## 2 Materials and methods

On 22 December 2021, we downloaded data from the Web of Science Core Collection (WoSCC); two authors independently verified citations and retrieved studies. The WoSCC is a frequently used authoritative database for scientific information, from which we generated a clustered network of 69,806 references cited by 1,754 studies. Between the publication years 2000 and 2021, literature searches were performed using the search terms: [TS = (“deep learning” OR “machine learning” OR “convolutional neural network*” OR CNN* OR RNN OR “Recurrent neural network*” OR “Fully Convolutional Network*” OR FCN*)], and The literature type = “Article OR Review OR Opening Online”, WoS category = Genetic heredity. Information on the following topics was collected: title, abstract, authors, institution, country/region, journal, keywords, and references. Articles were indexed in the WoSCC and excluded meeting articles, repeated articles, proceedings articles, book chapters, and unpublished documents without enough information for further analysis at the same time.

We described publication characteristics, including institutes, countries, journals, and keywords. The Journal of Citation Reports (JCR, 2021 version) was accessed to identify impact factors that reflected the scientific value of research (Eyre-Walker and Stoletzki, 2013). Retrieved data were analyzed in VOS viewer (Leiden University, Leiden, Holland) and CiteSpace V (Drexel University, Philadelphia, PA, United States), which facilitated collaborative network analyses connecting different publication characteristics (Chen, 2006; Chen, 2017). From the analysis and measures above, we obtain the current characteristics, research hotspot, subject evolution path, and future trajectories in deep learning applications of genetics.

## 3 Results

### 3.1 Distribution of articles by publication years

**FIGURE 1:**
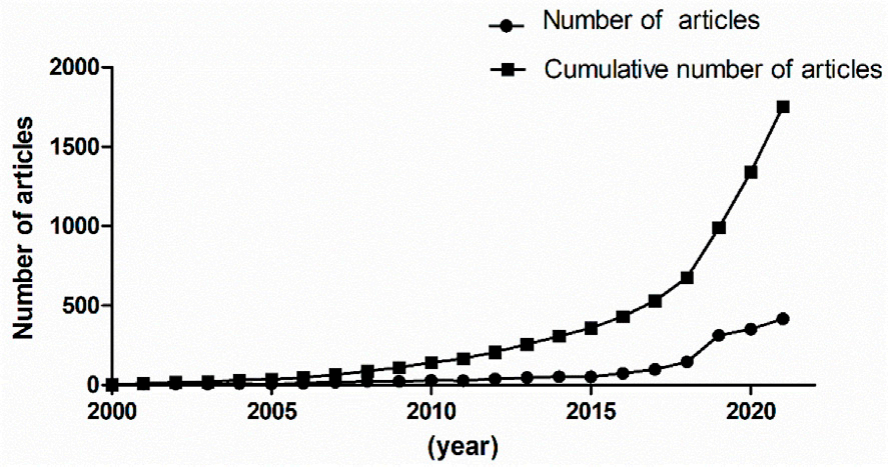
Number of published articles about deep learning application in genetics research from 2000 to 2021.

A total of 1,754 articles from 2000 to 2021 were published. As shown in Figure 1, the line with points denoted by square shows the trend of publications from 2000 to 2021, and the line with points denoted by circles shows the number of articles published each year. The number of published articles showed a rapid increase since 2018, and more than 70% of the total articles were published in the last 4 years. This suggests that the studies of deep learning applied in genetics research were new research hot points in recent years.

### 3.2 Analysis of countries, institutions and journals

**FIGURE 2:**
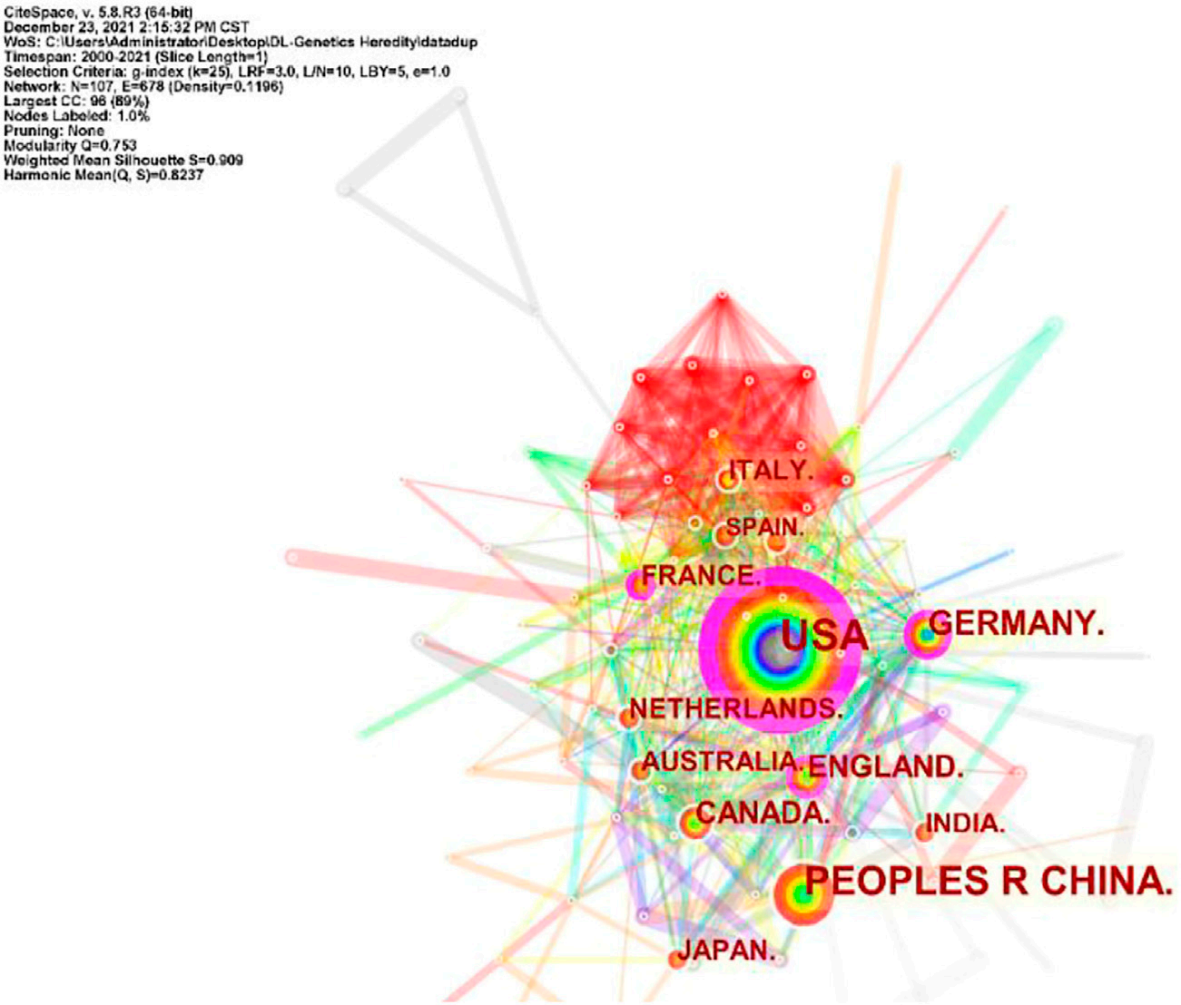
Cooperation of countries in the field of Deep Learning application in genetics research from 2000 to 2021.

**FIGURE 3:**
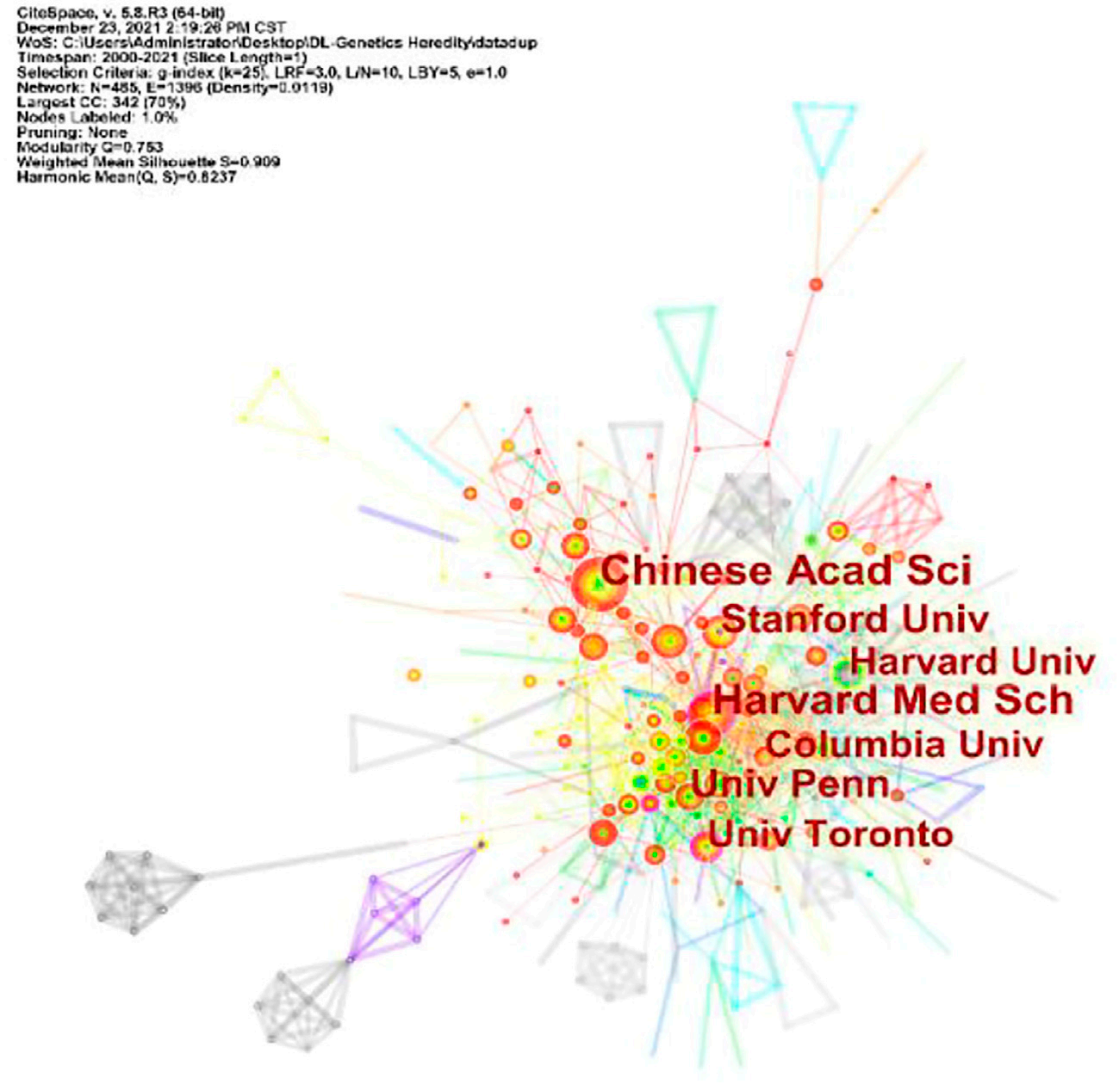
Cooperation of institutions contributed to publications for Deep Learning applications in genetic research.

A total of 101 countries and 2,487 institutes contributed publications. The top 10 countries, institutions, and cited journals are listed in Table 1. 728 (41.5%) articles published in the United States ranked first place, which was 18.3% higher than those in China, whose publication number was 407 (23.2%), thereby ranking second. However, it is worth noting that the research institution with the largest number (41 articles) of published articles was the Chinese Academy of Sciences, which indicated this institution had powerful scientific research ability in the field of deep learning application in genetics research. The collaborations between different countries and institutions are shown in Figures 2, 3. The bigger size of the circle represents the larger number of articles published by this country. The shorter the distance between two circles, the better the cooperation between the two countries. As shown in Figure 2, the biggest circle belongs to the United States of America which had close cooperation with Germany, England, and France. Although the Chinese Academy of Sciences published the largest number of articles, it was lack of cooperation with other institutions. Harvard Medical School was in a key position in this study field, which kept close cooperation with multiple institutions, such as Columbia University and Stanford University (shown in Figure 3).

**TABLE 1:** Top 10 countries, institutions, and journals.

Rank	Country	Count	H-index	Institution	Count	H-index	Cited journal	Count	If (2021)
1	United States	728	65	Chinese Academy of Sciences	41	13	BIOINFORMATICS	1,175	6.93
2	CHINA	407	35	Harvard Medical School	37	12	NATURE	1,082	49.96
3	GERMANY	118	33	Stanford University	29	18	NUCLEIC ACIDS RES	1,075	16.97
4	ENGLAND	101	35	University of Pennsylvania	28	11	P NATL ACAD SCI United States	868	11.20
5	CANADA	92	22	Harvard University	26	25	PLOS ONE	856	3.24
6	AUSTRALIA	54	19	University of Toronto	25	12	SCIENCE	780	47.72
7	INDIA	48	11	Columbia University	25	11	BMC BIOINFORMATICS	764	3.16
8	FRANCE	43	16	Yale University	24	14	NAT GENET	734	38.33
9	ITALY	41	15	University of Washington	24	13	CELL	683	41.58
10	JAPAN	41	16	Shanghai Jiao Tong University	22	9	GENOME BIOL	622	13.58

### 3.3 Journal analysis

**FIGURE 4:**
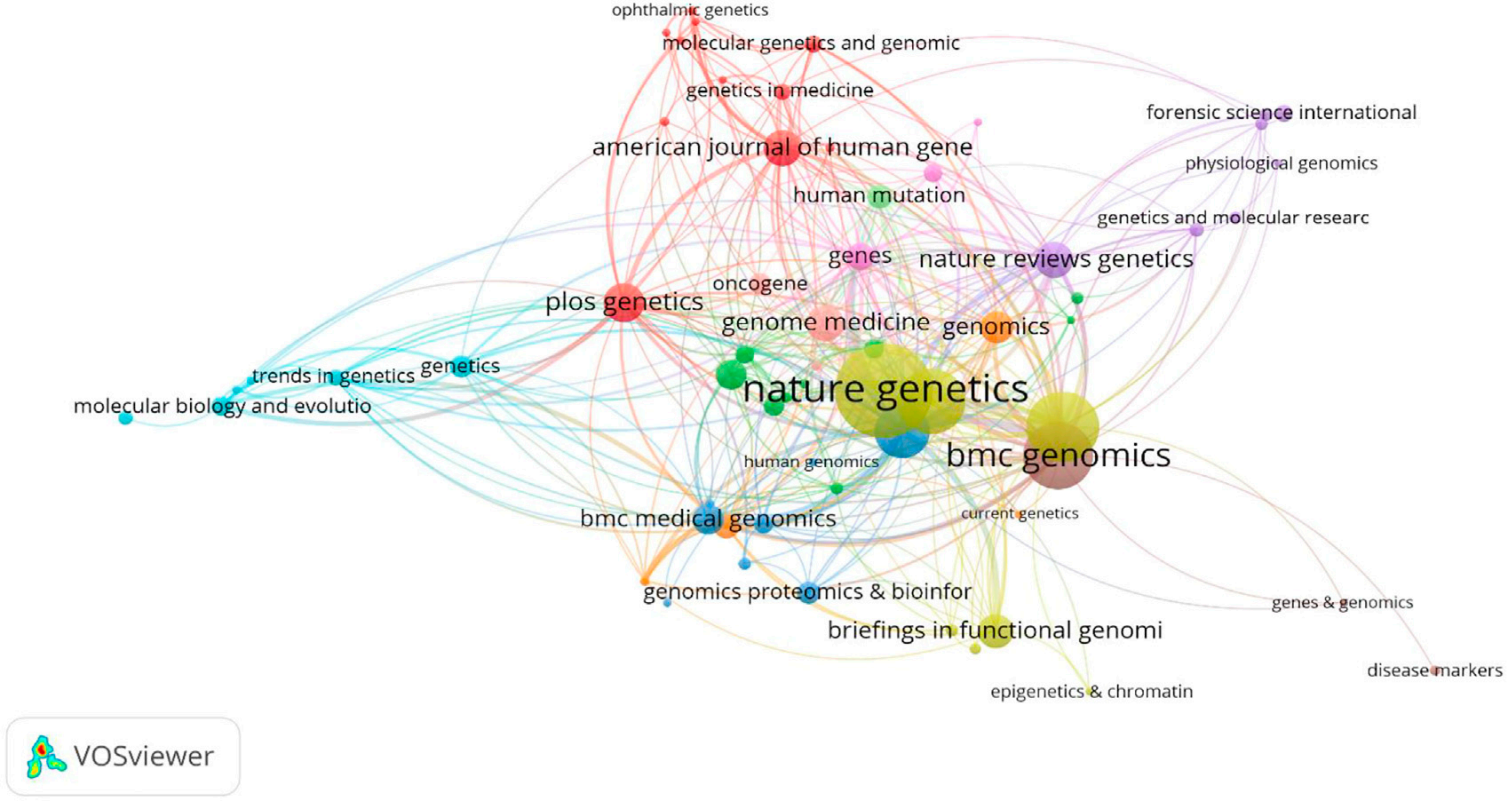
Network map of cited journals in the field of Deep Learning application in genetics research from 2000 to 2021.

A total of 151 cited journals published publications related to deep learning in genetics research. The top 10 cited journals are presented in Table 1(with green background). The highest cited count belonged to the BIOINFORMATICS (1,175 times), followed by NATURE (1,082 times). Among these journals, NATURE had the highest impact factor (49.962). Collaborations among these cited journals are shown in Figure 4.

**FIGURE 5:**
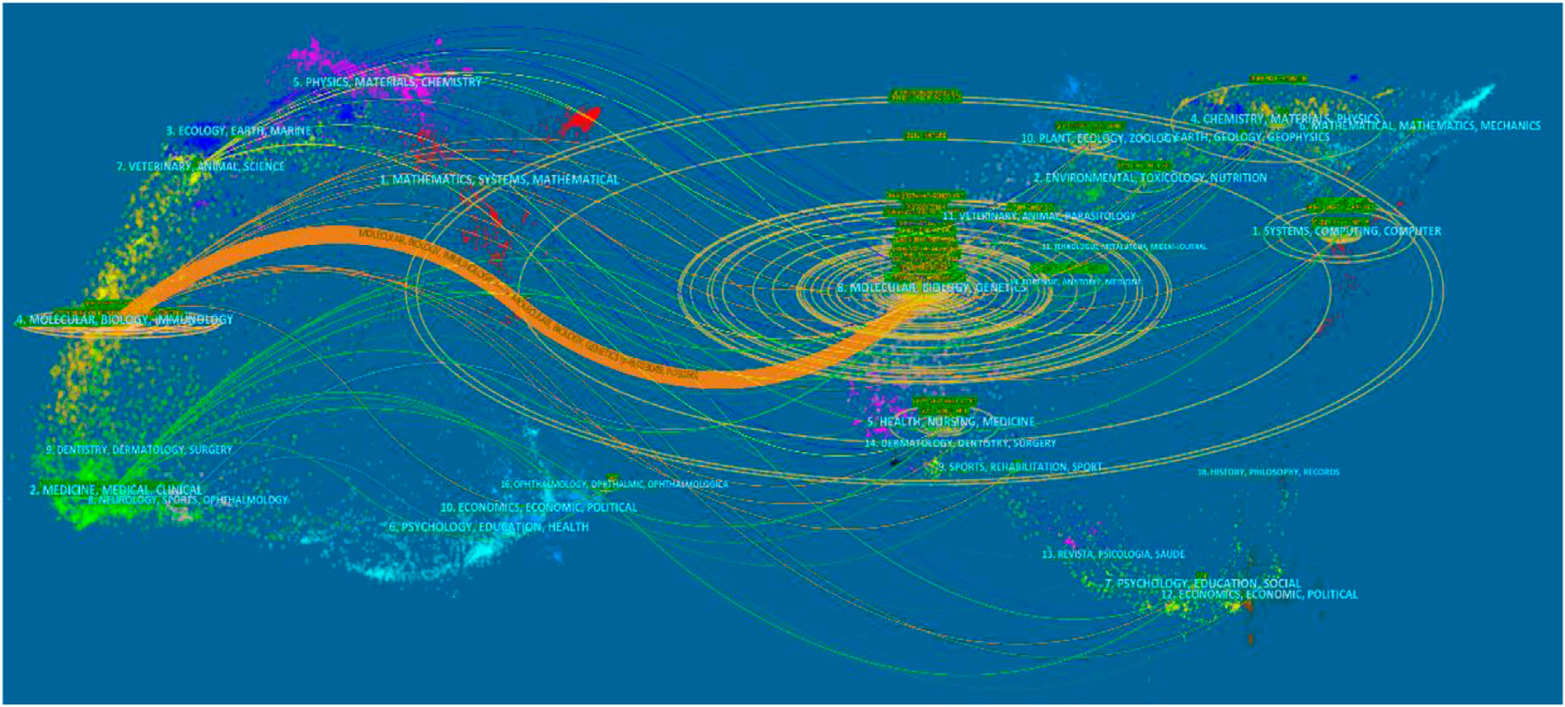
Dual-map overlay of journals in the field of Deep Learning application in genetics research from 2000 to 2021.

In academic journals, referential relationships facilitate knowledge exchange within the research field, where citing articles form the knowledge frontier and cited articles form the knowledge base. A journal dual-map overlay is shown in Figure 5. The cluster analysis of citing articles (the left side) belongs to journals focusing on the field of molecular/biology/immunology research. Also, the cluster analysis of cited articles (the right side) belongs to journals focusing on the field of molecular/biology/genetics research. The primary citation path colored orange represents the citation relationship between the two clusters, which indicated that based on genetics research, deep learning tends to be applied to immunology.

### 3.4 Reference analyses

References are key bibliometric indicators as frequently cited documents can greatly influence their research areas (Table 2). The article was published on Nature which was cited 89 times, ranking first. Summarizing the highly cited topics, the result indicated that deep learning methods such as deep convolutional nets and recurrent nets have dramatically improved drug discovery and genomics research.

**TABLE 2:** Top 10 cited references on VR in rehabilitation.

Rank	DOI	Title of cited reference	Count	Centrality	Interpretation of the findings	Year
1	10.1038/nature14539	Deep learning	89	0.01	This article discussed deep learning methods such as deep convolutional nets and recurrent nets that have dramatically improved speech and visual recognition. Other domains such as drug discovery and genomics brought about breakthroughs	2015
2	10.1038/nbt.3300	Predicting the sequence specificities of DNA- and RNA-binding proteins by deep learning	77	0.08	This study built a stand-alone software by using a diverse array of experimental data and evaluation metrics ascertained sequence specificities that is essential for identifying causal disease variants	2015
3	10.1038/nmeth.3547	Predicting effects of noncoding variants with a deep learning-based sequence model	65	0.07	This document developed a deep learning–based algorithmic framework that enables the prediction of noncoding variants	2015
4	10.1145/2939672.2939785	Proceedings of the 22nd ACM SIGKDD International Conference on Knowledge Discovery and Data Mining	54	0	This study described a highly effective scalable tree boosting machine learning method and proposes a novel sparsity-aware algorithm for sparse data and weighted quantile sketch for approximate tree learning	2016
5	10.1101/gr.200535.115	Basset: learning the regulatory code of the accessible genome with deep convolutional neural networks	47	0.1	This study offered a powerful computational approach to annotating and interpreting the noncoding genome. Researchers perform a single sequencing by CNN's assay to annotate every mutation in the genome with its influence on present accessibility and latent potential for accessibility	2016
6	10.1038/nature19057	Analysis of protein-coding genetic variation in 60,706 humans	44	0.01	This study analysis protein-coding genetic variation in 60,706 humans, and it can efficiently filtering of candidate disease-causing variants and discover human ‘knockout’ variants in protein-coding genes	2016
7	10.1093/nar/gkw226	DanQ: a hybrid convolutional and recurrent deep neural network for quantifying the function of DNA sequences	36	0.05	This study proposed a novel hybrid convolutional and bi-directional long short-term memory recurrent neural network framework for predicting non-coding function *de novo* from the sequence	2014
8	10.1038/nature14248	Integrative analysis of 111 reference human epigenomes	36	0.08	The article described the integrative analysis of 111 reference human epigenomes generated and profiled for histone modification patterns, DNA accessibility, DNA methylation, and RNA expression	2015
9	10.15252/msb.20156651	Deep learning for computational biology	34	0.03	This study reviewed the applications of this new breed of analysis approaches in regulatory genomics and cellular imaging	2014
10	10.1038/ng.2892	A general framework for estimating the relative pathogenicity of human genetic variants	34	0.05	This study discussed a framework that objectively integrates many diverse annotations into a single, quantitative score to differentiate 14.7 million simulated variants	2015

In network research, betweenness centrality is a major indicator to determine the importance of nodes in the network, and a higher betweenness centrality means that the literature is more important Synnestvedt et al., (2005). Table 2 also shows the betweenness centrality of these works of literature.

**FIGURE 6:**
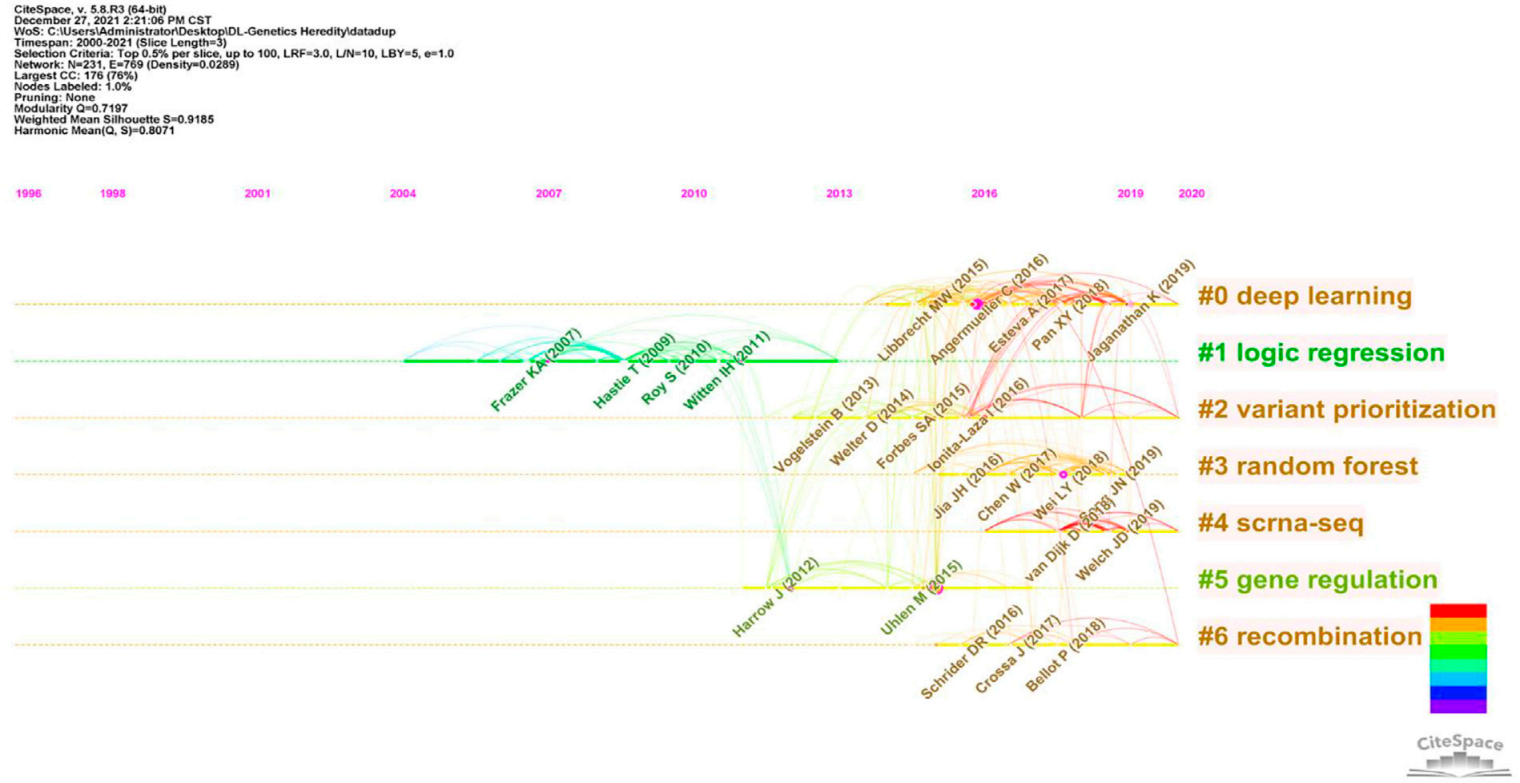
Reference co-citation map of publications from 2000 to 2021.

In this article, a co-cited document-based clustering analysis can be used to generate sub-fields and connect nodes in the research. We constructed a network of co-cited references to test the scientific relevance of related publications (Figure 6). Cluster setting parameters were Top N% = 0.5, #Years Per Slice = 3, and the pruning algorithm was chosen. The Modularity Q score = 0.7197, which was > 0.5, indicated the network adopted loosely coupled clusters. The Weighted Mean silhouette score = 0.9185, which was > 0.5, indicated acceptable cluster homogeneity. From the literature, we used index items as cluster markers (#0–#6); the largest cluster (#0) was “deep learning”, #1 was “logic regression”, #2 was “variant prioritization”, #3 was “random forests”, #4 was “scRNA-seq”, #5 was “genomic regulation”, and #6 was “recombination”.

### 3.5 Co-occurrence and burst keyword analyses

**FIGURE 7:**
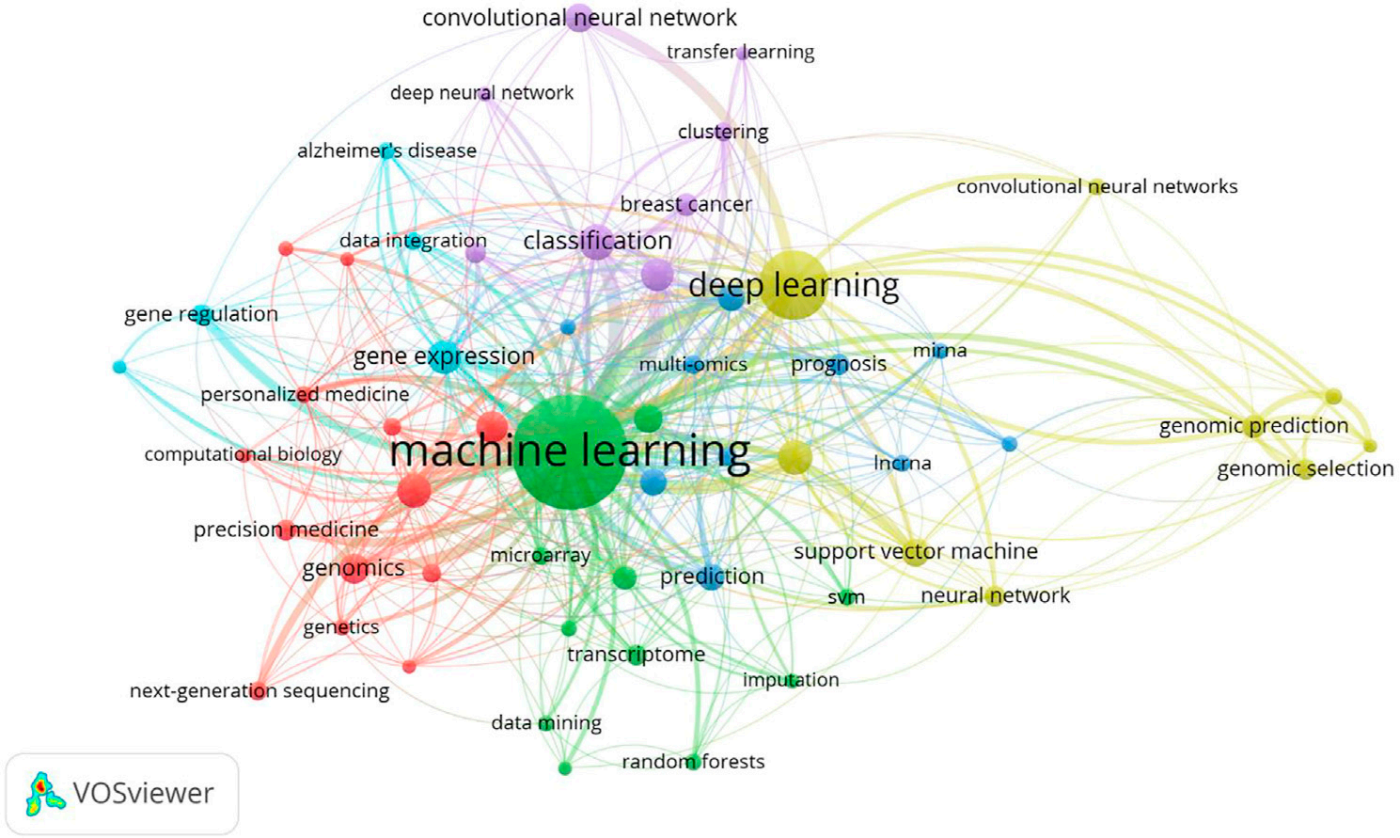
Network map of keywords is divided into 6 clusters.

**FIGURE 8:**
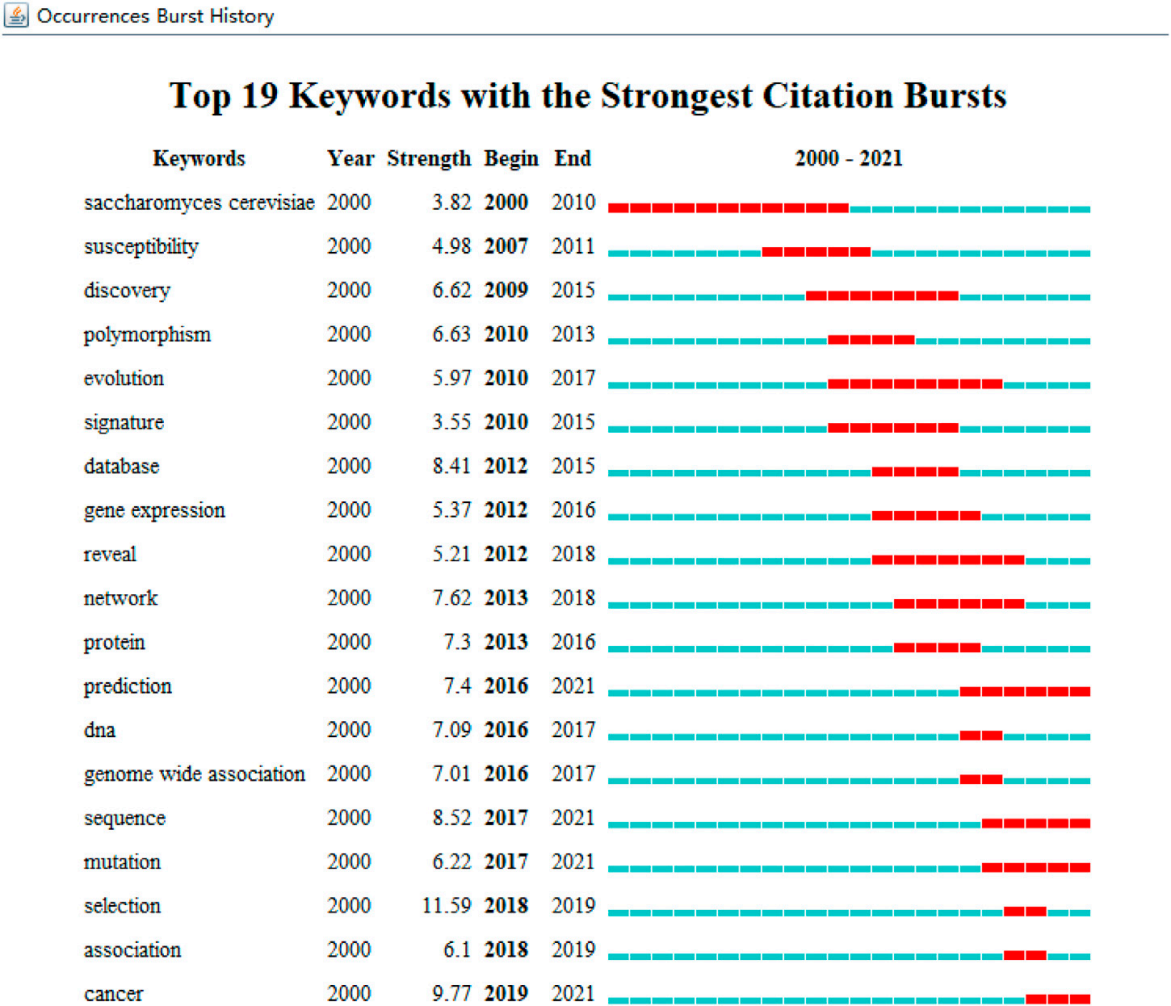
Keywords with the strongest citation bursts of publications from 2000 to 2021.

We extracted and analyzed keyword co-occurrence in related publications. The top 20, with highly linked strengths, are shown in Table 3. The co-occurrence of any two terms indicates their presence in the same publication. While identifying thematics in research areas, keyword analyses of articles 1754) identified 27 keywords with a minimum of 40 occurrences (Figure 7). The co-occurrence analysis based on author keywords was built with occurrence times as a threshold. There are several distinct clusters with different colors. The co-occurrence network map of keywords is shown in Figure 7, given that the larger the size of the circle, the higher the co-occurrence of keywords. Furthermore, having closer keywords together shows a stronger relationship. The average year of publication of the keywords was determined using colors. Machine learning, deep learning, and genetics constitute the largest circle of all keywords that are identified through co-occurrence analysis. Our study also investigated temporal trends in hotspot shifts using the top 19 keywords having the strongest citation bursts. These included prediction (2016–2021), sequence (2017–2021), mutation (2017–2021), and cancer (2019-2021) (Figure 8).

**TABLE 3:** Highly link strength of the top 20 occurrence keywords.

Rank	Keyword	Occurrence	Total link strength	Rank	Keyword	Occurrence	Total link strength
1	Machine learning	553	481	11	DNA methylation	36	44
2	Deep learning	201	194	12	Support vector machine	32	44
3	Classifications	54	90	13	Prediction	31	43
4	Random forest	52	69	14	Biomarker	30	45
5	Bioinformatics	48	68	15	Cancer	30	46
6	Gene expression	48	83	16	Rna-seq	24	38
7	Feature selection	46	80	17	Genomic prediction	23	42
8	Artificial intelligence	43	80	18	Breast cancer	22	32
9	Genomics	37	56	19	Gene regulation	19	26
10	Convolutional neural	36	24	20	Neural network	19	24

## 4 Discussion

### 4.1 General data

Between the publication years 2000–2021, we selected and investigated 1754 SCIE articles related to deep learning in genetics. Since 2015 with the development of gene sequencing technology, biological genetic data have exploded. The number of published articles showed a rapid increase. Another growth time node is 2018, more than 70% of the total articles were published in the last 4 years. This suggests that as deep learning technology enters its mature stage, it has attracted widespread attention. The highest number of studies (728) was generated by the United States, with China in second place at 407. The top ten institutions included seven in the United States and two in China. According to our data, most of the research in deep learning in genetics was produced by institutions and countries in developed countries, such as United States, Germany, and France. The reason for this trend is that better socioeconomic development can be the premise of ensuring adequate funding, resources, and human input to explore brand new scientific research. Socioeconomic factors such as GDP, GDP per capita, research and development funding, number of researchers, number of physicians, or international collaboration are important decisive factors of scientific productivity. There are many reasons for this trend, such as GDP level Nature Genetics was the most frequently used publishing journal; therefore, it significantly contributed to research in this area. Additionally, we investigated the top 10 cited publications; the top-cited article was published by Lec et al. on Nature and was cited 89 times. These high cited articles will shed some light to this research field.

### 4.2 The knowledge base and current research characteristics

In previous studies, different deep learning research applications have been investigated in genetics and generated significant results. As indicated (Figure 6), after clustering co-cited references, key clustering nodes successfully identified knowledge bases, namely: #0 “deep learning”, #1 “logic regression”, #2 was marked as “variant prioritization”, #3 was marked as “random forests”, cluster #4 was marked as “scrna-seq”, cluster #5 was marked as “genomic regulation”, and cluster #6 was marked as “recombination”. We described the knowledge base according to different clusters with time characteristics.

In the #0 “deep learning” cluster, applications of deep learning methods show cutting-edge performance in a variety of complex prediction tasks and large datasets in natural images. Scientists propose a deep-learning framework for genetic research events, e.g., distant metastasis in cancer, protein subcellular localization, genome recombination map of African *Drosophila melanogaster*, and DNA transcription factor binding; the abovementioned aspects show the advantages (Xiao et al., 2019; Adrion et al., 2020; Zhang et al., 2021a; Chereda et al., 2021).

In the #1 “logic regression” cluster, Liu et al. proposed a logic regression-based approach that was used to analyze the gene–gene interaction of eight genes involved in cell adhesion in 806 NSCL/P Chinese case-parent triad recruited to explore the risk of non-syndromic cleft lip Liu et al., (2019). Nicodemus KK’s team tested and discussed the interactions between these susceptibility genes using four machine learning algorithms (including random forest, generalized enhanced regression, and Monte Carlo logistic regression) in a case-control study of schizophrenia Nicodemus et al., (2010). Dasgupta et al. reviewed machine learning and regression-based methods in 200 common or rare genetic variants from exome sequencing data and discussed cross-validation for model assessment and selection Dasgupta et al., (2011).

In the #2 “variant prioritization” cluster, key challenges in genomics research are variant prioritization methods. Huang et al. identified a deep learning framework, which was evolution-based, for unified variant and gene prioritization. The authors integrated constraints predicting missense variants and protein-coding genes associated with dominant disorders and estimated fitness effects for potential single-nucleotide variants, which outperformed current methods Huang, (2020). Zhang et al. formulated a disease-specific variant classifier that assessed discriminate pathogenic variants from benign variants and prioritized disease-associated variants Zhang et al., (2021b). In their study, Mattia et al. proposed an automated computational framework that identified causal genetic variants (small insertions and deletions and coding/splicing single-nucleotide variants) to improve causal variant prioritization methods and variant pathogenicity classifications Bosio et al., (2019).

For the #3 “random forest” cluster, as a standard regression model, which has been widely used in the machine learning (ML) application, Jian Y’s team applied a random forest machine learning algorithm to purity pediatric children central nervous system tumor analysis, which helps with the clinical management of pediatrics Yang et al., ( 2021). Chen Z constructed a deep learning network model based on the random forest classifier, and it can easily identify malonylation sites, for predicting sites shows high confidence Chen et al., (2018). Nicholls et al. reviewed ML model (gradient boosting and random forests) applications, dissected variant and gene signal heterogeneity, prioritized complex disease-associated loci, and critically evaluated prioritization issues for genome-wide association investigations Nicholls et al., (2020).

For the #4 “scRNA-seq” cluster, as single-cell RNA-sequencing (scRNA-seq) is used to analyze gene expression with high resolution, scientists have comprehensively exploited this area to dissect individual cell types in several diseases. For example, Carlos et al. generated a Deep Neural Network (DNN) model which quantified immune infiltration levels in breast and colorectal cancer bulk RNA-seq samples and identified improved and accurate survival prediction and quantification data (Torroja and Sanchez-CaboDigitaldlsorter, 2019). Cédric et al., in an effort to accommodate increasing levels of scRNA-seq data, designed a deep neural network–based imputation algorithm that is more suitable for the ever-increasing scRNA-seq data (Arisdakessian et al., 2019). Additionally, Yao and Nelson’s teams generated unsupervised deep learning methods for improved data integration which showed improved performances in scRNA-seq datasets (Johansen and Quon, 2019; He et al., 2020).

For the #5 “gene regulation” cluster, an ML modality was adapted by Colbran et al. to impute gene regulation information from genotype data and investigate 490 ancient Eurasian human DNA samples and explore divergent gene regulation mechanisms which contributed to skin pigmentation and metabolic and immune functions. The authors identified gene regulation roles in adaptation and associations between complex traits and genetic diversity Colbran et al., (2021). Atak et al. devised a deep learning approach and integrative genomics strategy to analyze functional enhancer mutations with allelic imbalance of gene expression and chromatin accessibility and successfully interpreted and predicted the impact of a mutation on gene regulation Atak et al., (2021). Godwin et al. devised a deep learning–based model to predict the gene regulatory effects of low-molecular-weight compounds; the model potentially identified drug candidates inducing particular gene responses, without prior interactional information on protein targets Woo et al., (2020).

For the #6 “recombination” cluster, a central tenet of genomics is the accurate assessment of genome-wide recombination rates in natural populations. Andrew *et al.* used ML algorithms to examine if DNA motifs across the genome could be used to predict crossover variation and identify genetic factors influencing variation in recombination rates Adrian et al., (2016). Kha F proposed a DL intelligent computational predictor based on the deep neural network (DNN) as a classification engine for the identification of recombination spots through an experimental benchmark dataset with 10-fold cross-validation which achieved the 95.81% highest accuracy Khan et al., (2020).

### 4.3 Hotspots and frontiers in research

Keywords concentrate on contemporary research issues or concepts, while burst keywords represent emerging trends and frontiers in research. In our work, we used CiteSpace to capture burst keywords, and four related research frontiers were identified: four keywords with the strongest citation bursts, such as prediction (2016–2021), sequence (2017–2021), mutation (2017–2021), cancer (2019–2021), and these key words cover the research frontier of the current topic.

#### 4.3.1 Sequence (2017–2021)

Large-scale genetic datasets and deep-learning approaches are increasingly exploited by bioinformatics approaches to model protein structures and complexes. Zhao et al., using sequence information*,* devised a deep forest-based protein location algorithm to accurately predict protein subcellular locations using only protein sequences, which outperformed contemporary state-of-art algorithms Zhao et al., (2018). Cui et al. analyzed the main methods used to represent protein sequence data, theoretically reviewed the architecture of different embedding models, and investigated the development of these sequence-embedding approaches Cui et al., (2021). Braberg et al. analyzed the emergence of large-scale genetic datasets and deep learning approaches which modeled protein structures and associated interactions (deep mutational scanning, genome-scale genetic or chemical-genetic interaction mapping, and coevolution) and discussed structural data integration from different sources Braberg et al., (2022).

#### 4.3.2 Cancer (2019–2021)

Originally used for image processing and pattern recognition methods, deep learning models are now used to detect genetic alterations in cancer and determine cancer patient prognoses. The framework by Mallik et al. integrated linear regression, differential expression, and deep learning and facilitated the robust interpretation of DNA methylation signatures and gene expression data for cervical cancer Mallik et al., (2020). Poirion et al., using multi-omics data*,* established a deep learning ensemble network that predicted patient survival subtypes Poirion et al., (2021)^.^ In order to predict survival outcomes in cancer patients, Huang et al. broadly analyzed The Cancer Genome Atlas cancers using several deep learning–based models Huang et al., (2020). Tran et al. reviewed emerging deep learning approaches and how they were applied to precision oncology. The authors not only exemplified how deep learning was used for cancer diagnostics, prognostics, and treatment management strategies, but they also reviewed the current limitations and challenges of deep learning in this area Vaernet, (1972).

#### 4.3.3 Mutation (2017–2021)

With considerable high-throughput technology advancements, somatic mutations in their millions have been reported, but critically, the identification of specific driver genes expressing oncogenic mutations is highly challenging and complex. In their study, Luo et al. used “deep drive” to predict driver genes by combining similarity networks with features that characterize the functional impact of mutations. They use AUC scores to evaluate predictive efficiency. DeepDriver achieved AUC scores of 0.984 and 0.976 on breast cancer and colorectal cancer, respectively, which were better than those of the competing algorithms. Luo et al., (2019) Sahraeian et al. inaugurated a deep convolutional neural network–based somatic mutation detection strategy using high-confidence somatic mutations in a cancer cell line. The authors generated comprehensive models using multiple datasets and highly robust and significantly superior methods when compared with traditional detection strategies Sahraeian et al., (2022).

#### 4.3.4 Prediction (2016–2021)

Ding YL provided a comprehensive review of ML-based approaches for predicting disease–biomolecule associations with multi-view data sources. They discussed feature representation methods and provided some perspectives for further improving biomolecule-disease prediction methods (Ding et al., 2021). Groschel MI presented a translational genomics platform for tuberculosis application to predict antibiotic resistance from next-generation sequence data. After benchmarking, it can rapidly and accurately predict resistance to anti-tuberculosis drugs Gröschel et al., (2021). Majumdar A et al. developed a novelty ensemble support vector regression to predict each drug response value for a single patient based on cell-line gene expression data. This can be used to develop a robust drug response prediction system for cancer patients using cancer cell lines guidance and multi-omics data (Majumdar et al., 2021).

## 5 Limitations

Our study still has some limitations to be addressed. First, we choose the SCIE database as the collection, while a few studies not included in the core collection were missed. Second, this study includes two types of publication (article and review), and the uneven quality of the collected publications may reduce the credibility of the mapping analysis. However, the visualized analysis based on bibliometric analysis undoubtedly lays a foundation for readers to quickly understand the research subjects, hotspots, and development trends in an unfamiliar research field.

## 6 Conclusions

Using bibliometrics, we systematically, comprehensively, and objectively investigated the literature related to deep learning applications in genetics research. Importantly, we identified research bases, current hotspots, and future trends in this area. The knowledge bases were “deep learning,” logic regression,” “variant prioritization,” “random forests,” “scRNA-seq,” “genomic regulation,” and “recombination”. We also provided hotspot and frontier guidance for researchers wishing to conduct advanced genetics research in the future. We identified research frontiers and emerging trends topics that incorporated prediction, sequence, mutation, and cancer. Finally, some studies selected for this research were not comprehensive and may have generated publication bias, thereby potentially affecting the study outcomes of this bibliometric review.

## Data Availability

The original contributions presented in the study are included in the article/Supplementary Material; further inquiries can be directed to the corresponding author.

## Abbreviations

DL, deep learning; ML, machine learning; CNNs, convolutional neural networks; DNN, deep neural network; scRNA-Seq, single-cell RNA sequencing; TCGA, The Cancer Genome Atlas; NSCL/P, nonsyndromic cleft lip with or without cleft palate.

## References

[B1] Adrian A. B. Corchado J. C. Comeron J. M. (2016). Predictive models of recombination rate variation across the *Drosophila melanogaster* genome. Genome Biol. Evol. 8, 2597–2612. 10.1093/gbe/evw181 27492232 PMC5010912

[B2] Adrion J. R. Galloway J. G. Kern A. D. (2020). Predicting the landscape of recombination using deep learning. Mol. Biol. Evol. 37, 1790–1808. 10.1093/molbev/msaa038 32077950 PMC7253213

[B3] Arisdakessian C. Poirion O. Yunits B. Zhu X. Garmire L. X. (2019). DeepImpute: An accurate, fast, and scalable deep neural network method to impute single-cell RNA-seq data. Genome Biol. 20, 211. 10.1186/s13059-019-1837-6 31627739 PMC6798445

[B4] Atak Z. K. Taskiran I. I. Demeulemeester J. Flerin C. Mauduit D. Minnoye L. (2021). Interpretation of allele-specific chromatin accessibility using cell state-aware deep learning. Genome Res. 31, 1082–1096. 10.1101/gr.260851.120 33832990 PMC8168584

[B5] Berrar D. Dubitzky W. (2021). Deep learning in bioinformatics and biomedicine. Brief. Bioinform. 22 (2), 1513–1514. 10.1093/bib/bbab087 33693457 PMC8485073

[B6] Bosio M. Drechsel O. Rahman R. Muyas F. Rabionet R. Bezdan D. (2019). eDiVA-Classification and prioritization of pathogenic variants for clinical diagnostics. Hum. Mutat. 40, 865–878. 10.1002/humu.23772 31026367 PMC6767450

[B7] Braberg H. Echeverria I. Kaake R. M. Sali A. Krogan N. J. (2022). From systems to structure - using genetic data to model protein structures. Nat. Rev. Genet. 23 (6), 342–354. 10.1038/s41576-021-00441-w 35013567 PMC8744059

[B8] Chen C. (2006). CiteSpace II: Detecting and visualizing emerging trends and transient patterns in scientific literature. J. Am. Soc. Inf. Sci. Technol. 57 (3), 359–377. 10.1002/asi.20317

[B9] Chen C. (2017). Science mapping: A systematic review of the literature. J. Data Inf. Sci. 2, 1–40. 10.1515/jdis-2017-0006

[B10] Chen Z. He N. Huang Y. Qin W. T. Liu X. Li L. (2018). Integration of A Deep learning classifier with A random forest approach for predicting malonylation sites. Genomics Proteomics Bioinforma. 16, 451–459. 10.1016/j.gpb.2018.08.004 PMC6411950 30639696

[B11] Chereda H. Bleckmann A. Menck K. Perera-Bel J. Stegmaier P. Auer F. (2021). Explaining decisions of graph convolutional neural networks: Patient-specific molecular subnetworks responsible for metastasis prediction in breast cancer. Genome Med. 13, 42. 10.1186/s13073-021-00845-7 33706810 PMC7953710

[B12] Colbran L. L. Johnson M. R. Mathieson I. Capra J. A. (2021). Tracing the evolution of human gene regulation and its association with shifts in environment. Genome Biol. Evol. 13 (11), evab237. 10.1093/gbe/evab237 34718543 PMC8576593

[B13] Cui F. Zhang Z. Zou Q. (2021). Sequence representation approaches for sequence-based protein prediction tasks that use deep learning. Brief. Funct. Genomics 20, 61–73. 10.1093/bfgp/elaa030 33527980

[B14] Dasgupta A. Sun Y. V. König I. R. Bailey-Wilson J. E. Malley J. D. (2011). Brief review of regression-based and machine learning methods in genetic epidemiology: The genetic analysis workshop 17 experience. Genet. Epidemiol. 35 (1), S5–S11. 10.1002/gepi.20642 22128059 PMC3345521

[B15] Ding Y. Lei X. Liao B. Wu F. X. (2021). Machine learning approaches for predicting biomolecule-disease associations. Brief. Funct. Genomics 20 (4), 273–287. 10.1093/bfgp/elab002 33554238

[B16] Esteva A. Robicquet A. Ramsundar B. Kuleshov V. DePristo M. Chou K. (2019). A guide to deep learning in healthcare. Nat. Med. 25 (1), 24–29. 10.1038/s41591-018-0316-z 30617335

[B17] Eyre-Walker A. Stoletzki N. (2013). The assessment of science: The relative merits of post-publication review, the impact factor, and the number of citations. PLoS Biol. 11, e1001675. 10.1371/journal.pbio.1001675 24115908 PMC3792863

[B18] Gröschel M. I. Owens M. Freschi L. Vargas R. Jr Marin M. G. Phelan J. (2021). GenTB: A user-friendly genome-based predictor for tuberculosis resistance powered by machine learning. Genome Med. 13 (1), 138. 10.1186/s13073-021-00953-4 34461978 PMC8407037

[B19] He Y. Yuan H. Wu C. Xie Z. (2020). DISC: A highly scalable and accurate inference of gene expression and structure for single-cell transcriptomes using semi-supervised deep learning. Genome Biol. 21, 170. 10.1186/s13059-020-02083-3 32650816 PMC7353747

[B20] Huang Y. F. (2020). Unified inference of missense variant effects and gene constraints in the human genome. PLoS Genet. 16, e1008922. 10.1371/journal.pgen.1008922 32667917 PMC7384676

[B21] Huang Z. Johnson T. S. Han Z. Helm B. Cao S. Zhang C. (2020). Deep learning-based cancer survival prognosis from RNA-seq data: Approaches and evaluations. BMC Med. Genomics 13, 41. 10.1186/s12920-020-0686-1 32241264 PMC7118823

[B22] Johansen N. Quon G. (2019). scAlign: a tool for alignment, integration, and rare cell identification from scRNA-seq data. Genome Biol. 20, 166. 10.1186/s13059-019-1766-4 31412909 PMC6693154

[B23] Khairi S. S. M. Bakar M. A. A. Alias M. A. Bakar S. A. Liong C.-Y. Rosli N. (2021). Deep learning on histopathology images for breast cancer classification: A bibliometric analysis. Healthcare 10, 10. 10.3390/healthcare10010010 35052174 PMC8775465

[B24] Khan F. Khan M. Iqbal N. Khan S. Muhammad Khan D. Khan A. (2020). Prediction of recombination spots using novel hybrid feature extraction method via deep learning approach. Front. Genet. 11, 539227. 10.3389/fgene.2020.539227 33093842 PMC7527634

[B25] Liu D. Wang M. Yuan Y. Schwender H. Wang H. Wang P. (2019). Gene-gene interaction among cell adhesion genes and risk of nonsyndromic cleft lip with or without cleft palate in Chinese case-parent trios. Mol. Genet. Genomic Med. 7, e00872. 10.1002/mgg3.872 31419083 PMC6785639

[B26] Luo P. Ding Y. Lei X. Wu F. X. (2019). deepDriver: Predicting cancer driver genes based on somatic mutations using deep convolutional neural networks. Front. Genet. 10, 13. 10.3389/fgene.2019.00013 30761181 PMC6361806

[B27] Majumdar A. Liu Y. Lu Y. Wu S. Cheng L. (2021). kESVR: An ensemble model for drug response prediction in precision medicine using cancer cell lines gene expression. Genes. 12 (6), 844. 10.3390/genes12060844 34070793 PMC8229729

[B28] Mallik S. Seth S. Bhadra T. Zhao Z. (2020). A linear regression and deep learning approach for detecting reliable genetic alterations in cancer using DNA methylation and gene expression data. Genes. (Basel), 11: 931. 10.3390/genes11080931 PMC7465138 32806782

[B29] Nicholls H. L. John C. R. Watson D. S. Munroe P. B. Barnes M. R. Cabrera C. P. (2020). Reaching the end-game for GWAS: Machine learning approaches for the prioritization of complex disease loci. Front. Genet. 11, 350. 10.3389/fgene.2020.00350 32351543 PMC7174742

[B30] Nicodemus K. K. Callicott J. H. Higier R. G. Luna A. Nixon D. C. Lipska B. K. (2010). Evidence of statistical epistasis between DISC1, CIT and NDEL1 impacting risk for schizophrenia: Biological validation with functional neuroimaging. Hum. Genet. 127, 441–452. 10.1007/s00439-009-0782-y 20084519

[B31] Poirion O. B. Jing Z. Chaudhary K. Huang S. Garmire L. X. (2021). DeepProg: An ensemble of deep-learning and machine-learning models for prognosis prediction using multi-omics data. Genome Med. 13, 112. 10.1186/s13073-021-00930-x 34261540 PMC8281595

[B32] Sahraeian S. Fang L. T. Karagiannis K. Moos M. Smith S. Santana-Quintero L. (2022). Achieving robust somatic mutation detection with deep learning models derived from reference data sets of a cancer sample. Genome Biol. 23 (1), 12. 10.1186/s13059-021-02592-9 34996510 PMC8740374

[B33] Synnestvedt M. B. Chen C. Holmes J. H. (2005). CiteSpace II: Visualization and knowledge discovery in bibliographic databases. AMIA Annu. Symp. Proc. 2005, 724–728. PMC1560567 16779135

[B34] Tang X. Shi Z. Jin M. (2021). Multi-category multi-state information ensemble-based classification method for precise diagnosis of three cancers. Neural Comput. Appl. 33, 15901–15917. 10.1007/s00521-021-06211-3

[B35] Torroja C. Sanchez-Cabo Digitaldlsorter F. (2019). Digitaldlsorter: Deep-Learning on scRNA-seq to deconvolute gene expression data. Front. Genet. 10, 978. 10.3389/fgene.2019.00978 31708961 PMC6824295

[B36] Tran K. A. Kondrashova O. Bradley A. Williams E. D. Pearson J. V. Waddell N. (2021). Deep learning in cancer diagnosis, prognosis and treatment selection. Genome Med. 13 (1), 152. 10.1186/s13073-021-00968-x 34579788 PMC8477474

[B37] Tripathi R. Patel S. Kumari V. Chakraborty P. Varadwaj P. K. (2016). DeepLNC, a long non-coding RNA prediction tool using deep neural network. Netw. Model. Anal. Health Inf. Bioinforma. 5, 21. 10.1007/s13721-016-0129-2

[B38] Vaernet K. (1972). Stereotaxic amygdalotomy in temporal lobe epilepsy. Stereotact. Funct. Neurosurg. 34, 176–183. 10.1159/000103055 4563548

[B39] Vellido A. (2020). The importance of interpretability and visualization in machine learning for applications in medicine and health care. Neural Comput. Appl. 32, 18069–18083. 10.1007/s00521-019-04051-w

[B40] Wang L. Wang H. Huang Y. Yan B. Chang Z. Liu Z. (2022) Trends in the application of deep learning networks in medical image. Eur. J. Radiology 146, 110069. 10.1016/j.ejrad.2021.110069 34847395

[B41] Woo G. Fernandez M. Hsing M. Lack N. A. Cavga A. D. Cherkasov A. DeepC. O. P. (2020). DeepCOP: Deep learning-based approach to predict gene regulating effects of small molecules. Bioinformatics 36, 813–818. 10.1093/bioinformatics/btz645 31504186

[B42] Wu S. Roberts K. Datta S. Du J. Ji Z. Si Y. (2020). Deep learning in clinical natural language processing: A methodical review. J. Am. Med. Inf. Assoc. 27 (3), 457–470. 10.1093/jamia/ocz200 PMC7025365 31794016

[B43] Xiao M. Shen X. Pan W. (2019). Application of deep convolutional neural networks in classification of protein subcellular localization with microscopy images. Genet. Epidemiol. 43, 330–341. 10.1002/gepi.22182 30614068 PMC6416075

[B44] Xie R. Wen J. Quitadamo A. Cheng J. Shi X. (2017). A deep auto-encoder model for gene expression prediction. BMC Genomics 18 (9), 845. 10.1186/s12864-017-4226-0 29219072 PMC5773895

[B45] Yang J. Wang J. Tian S. Wang Q. Zhao Y. Wang B. (2021). An integrated analysis of tumor purity of common central nervous system tumors in children based on machine learning methods. Front. Genet. 12, 707802. 10.3389/fgene.2021.707802 34925437 PMC8678112

[B46] Zhang X. Walsh R. Whiffin N. Buchan R. Midwinter W. Wilk A. (2021). Disease-specific variant pathogenicity prediction significantly improves variant interpretation in inherited cardiac conditions. Genet. Med. 23, 69–79. 10.1038/s41436-020-00972-3 33046849 PMC7790749

[B47] Zhang Y. Mo Q. Xue L. Luo J. (2021). Evaluation of deep learning approaches for modeling transcription factor sequence specificity. Genomics 113, 3774–3781. 10.1016/j.ygeno.2021.09.009 34534646

[B48] Zhao L. Wang J. Nabil M. M. Zhang J. (2018). Deep forest-based prediction of protein subcellular localization. Curr. Gene Ther. 18, 268–274. 10.2174/1566523218666180913110949 30209998

[B49] Zou J. Huss M. Abid A. Mohammadi P. Torkamani A. Telenti A. (2019). A primer on deep learning in genomics. Nat. Genet. 51, 12–18. 10.1038/s41588-018-0295-5 30478442 PMC11180539

